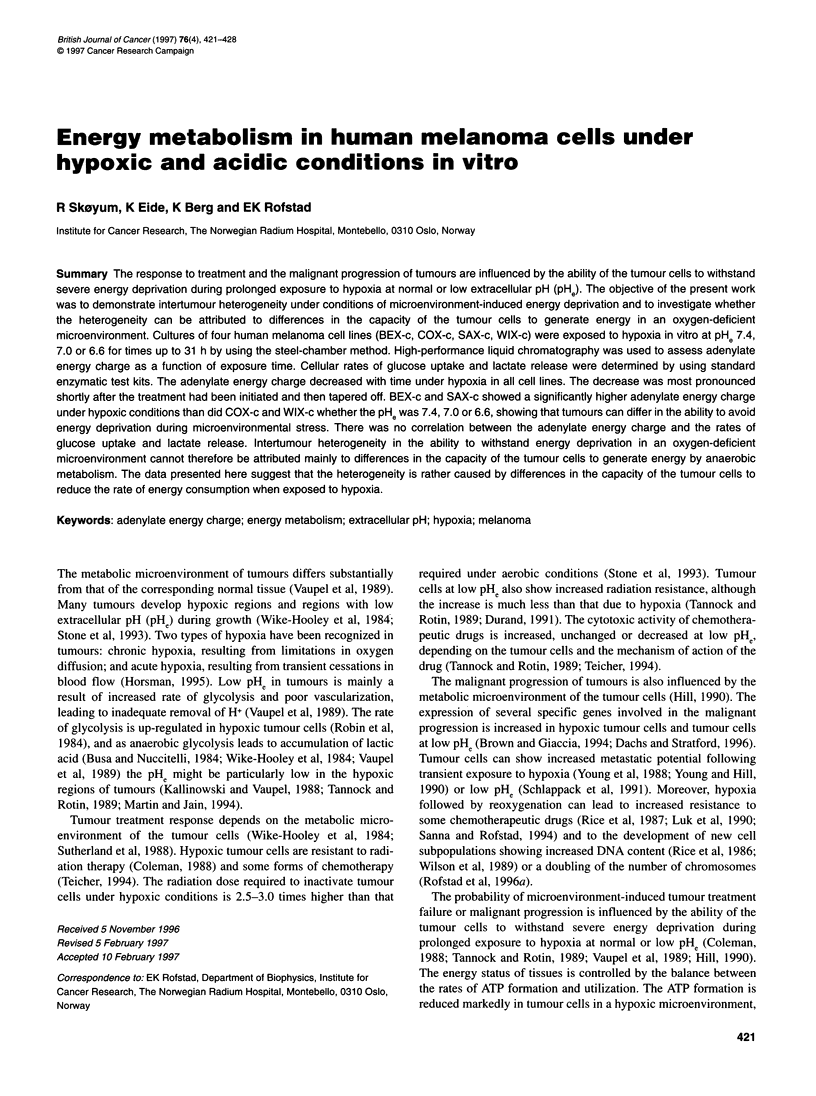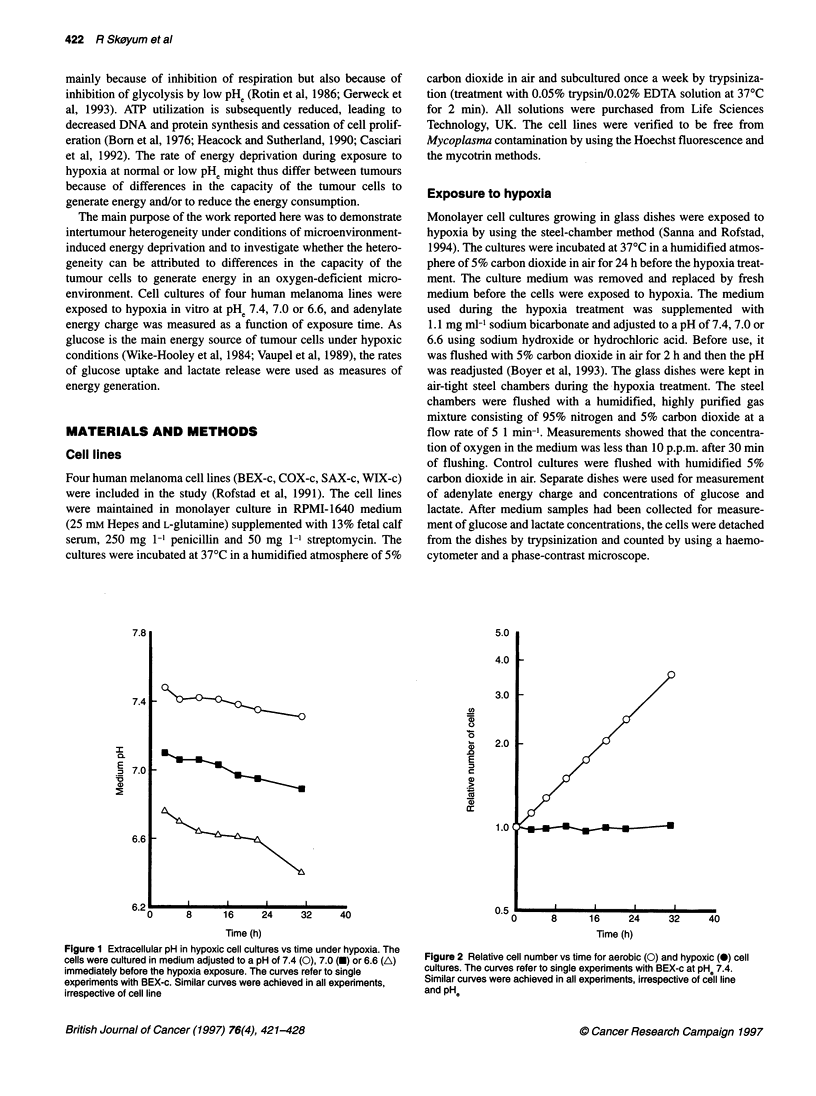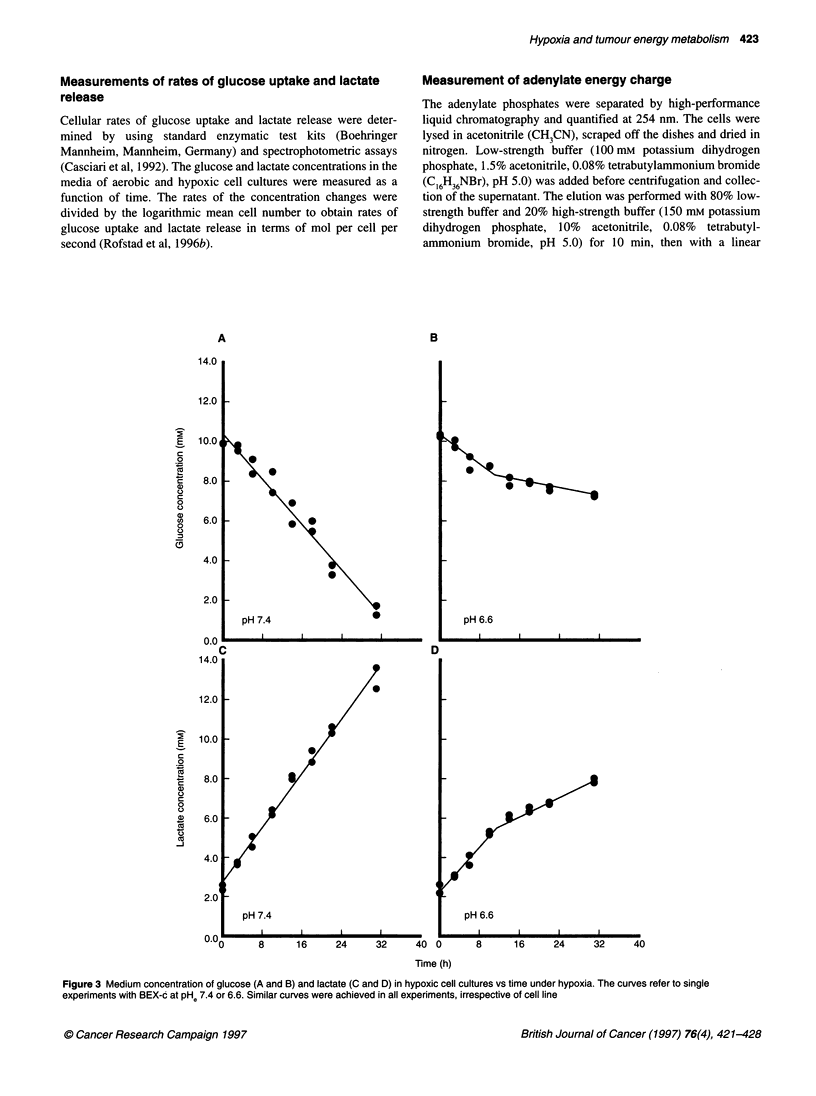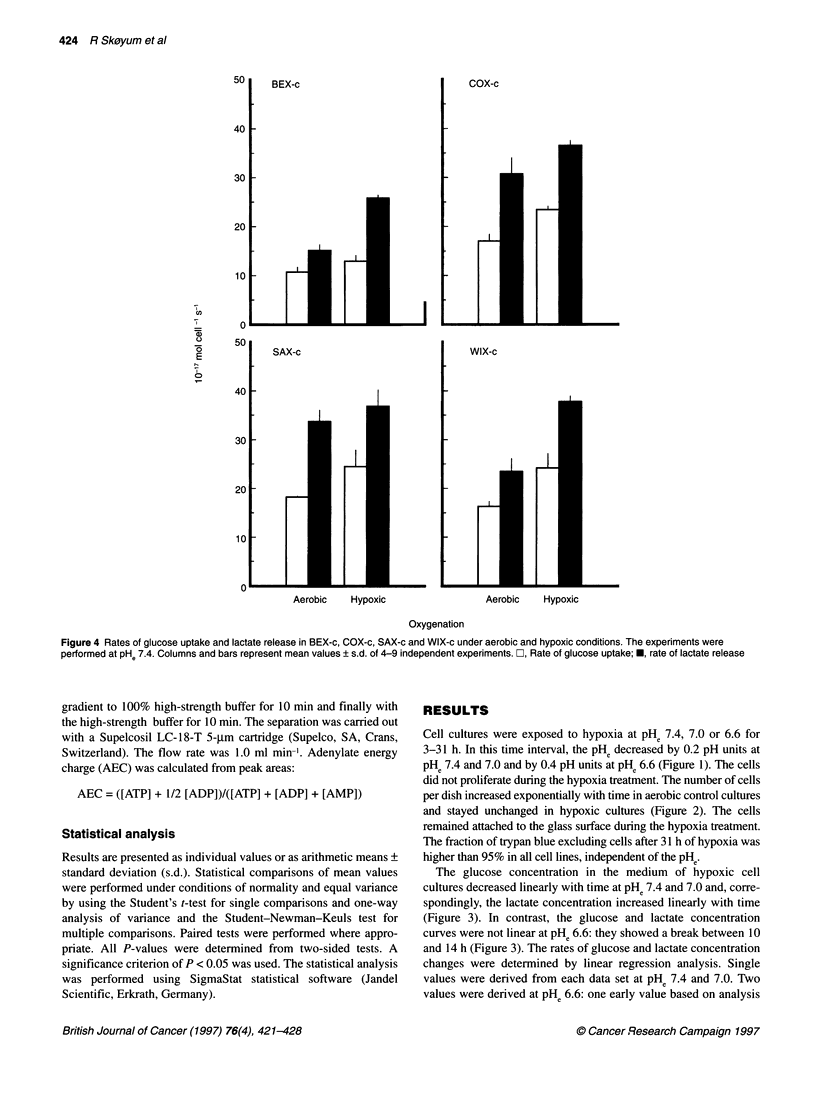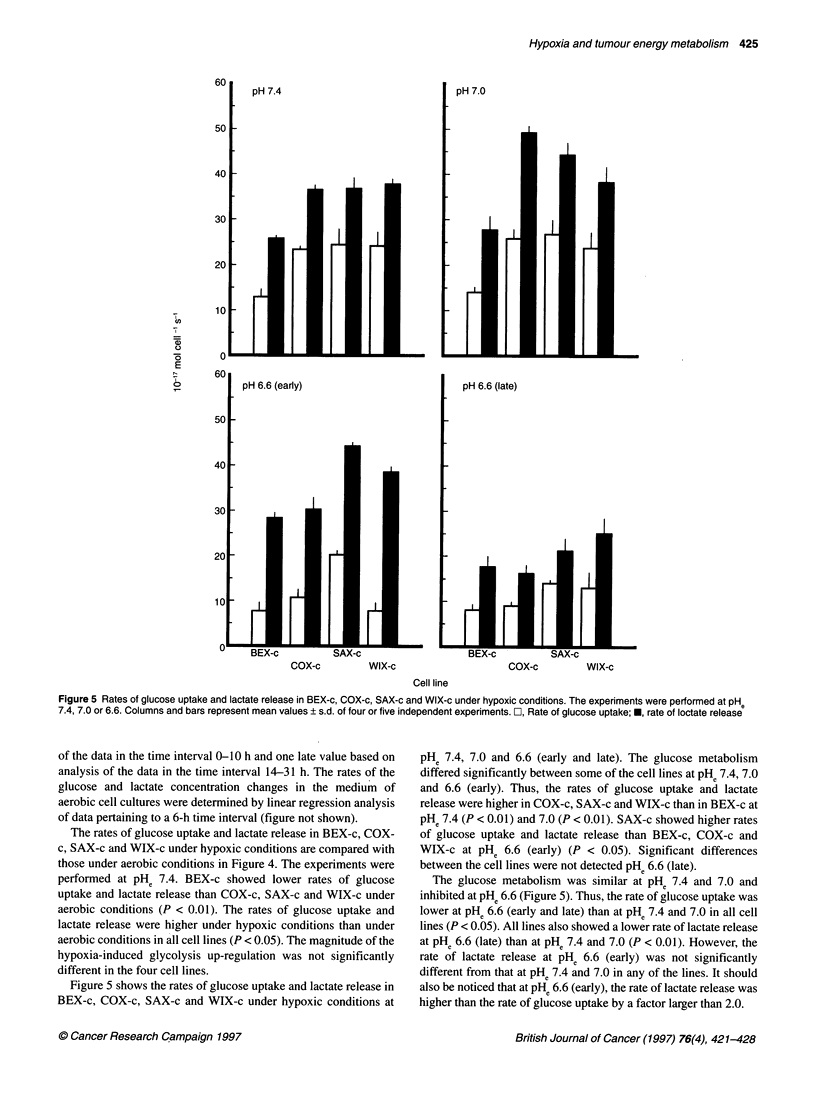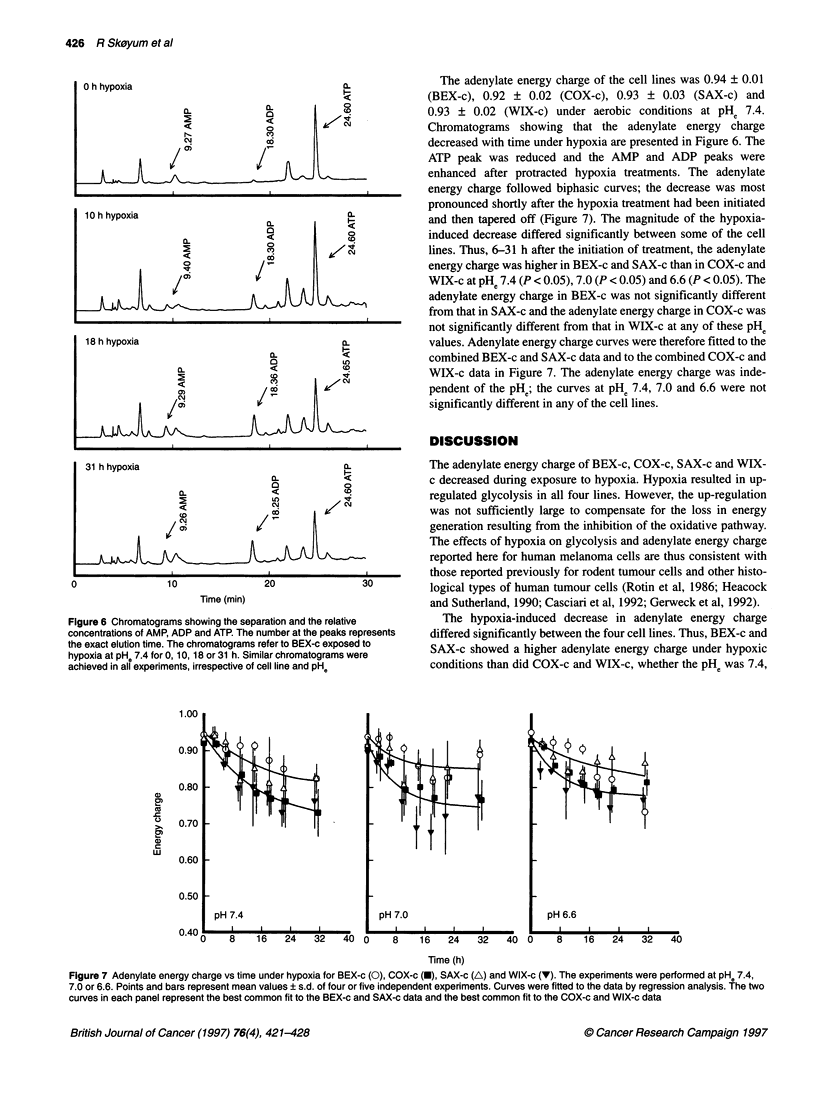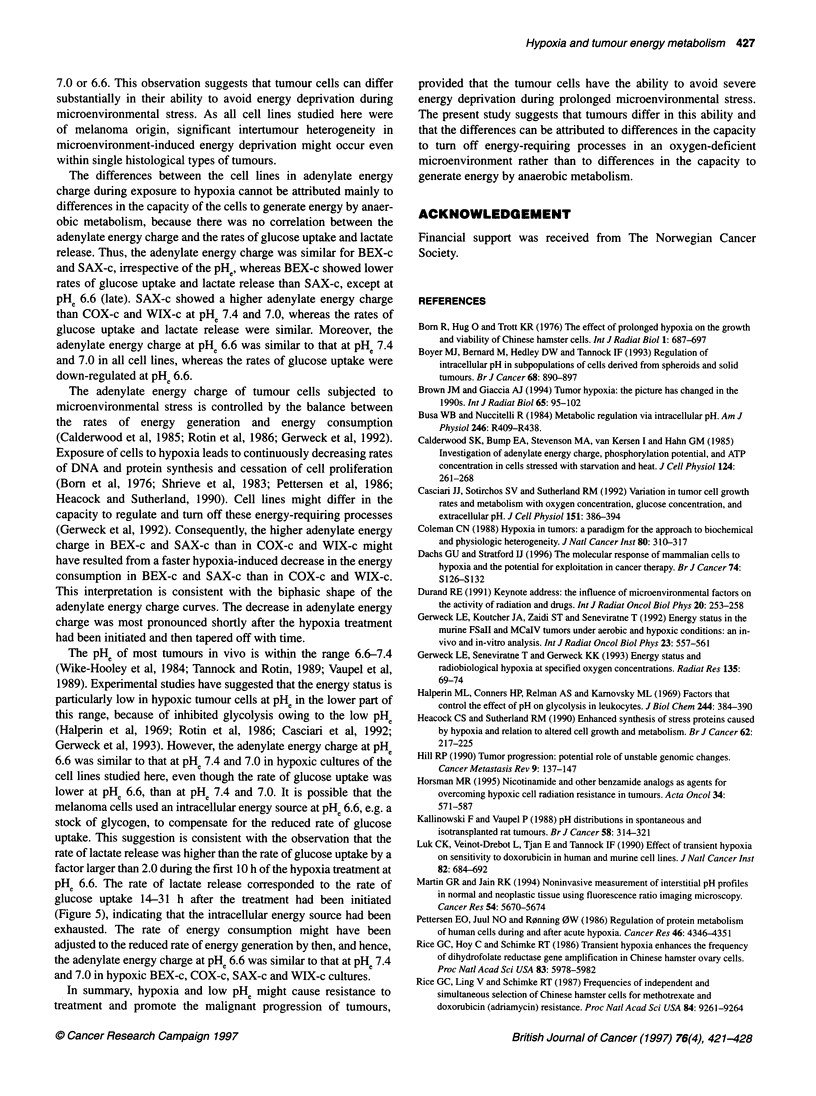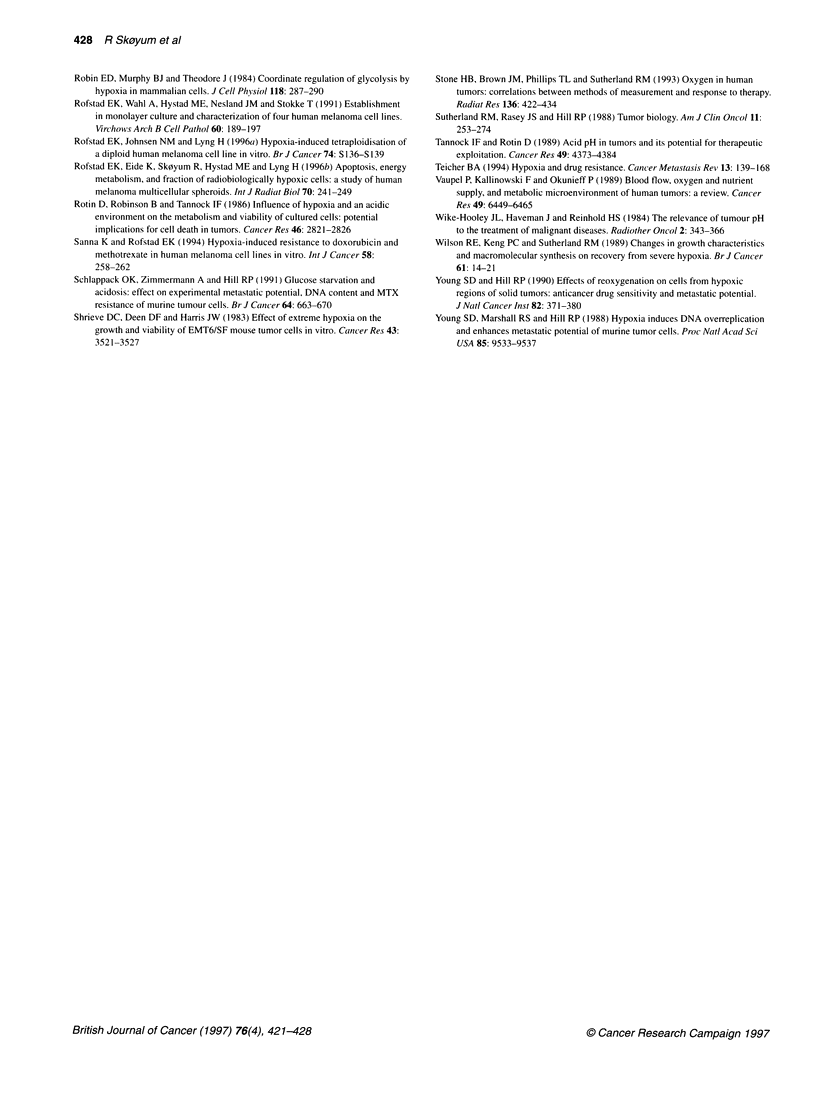# Energy metabolism in human melanoma cells under hypoxic and acidic conditions in vitro.

**DOI:** 10.1038/bjc.1997.405

**Published:** 1997

**Authors:** R. SkÃ¸yum, K. Eide, K. Berg, E. K. Rofstad

**Affiliations:** Institute for Cancer Research, The Norwegian Radium Hospital, Montebello, Oslo, Norway.

## Abstract

The response to treatment and the malignant progression of tumours are influenced by the ability of the tumour cells to withstand severe energy deprivation during prolonged exposure to hypoxia at normal or low extracellular pH (pHe). The objective of the present work was to demonstrate intertumour heterogeneity under conditions of microenvironment-induced energy deprivation and to investigate whether the heterogeneity can be attributed to differences in the capacity of the tumour cells to generate energy in an oxygen-deficient microenvironment. Cultures of four human melanoma cell lines (BEX-c, COX-c, SAX-c, WIX-c) were exposed to hypoxia in vitro at pHe 7.4, 7.0 or 6.6 for times up to 31 h by using the steel-chamber method. High-performance liquid chromatography was used to assess adenylate energy charge as a function of exposure time. Cellular rates of glucose uptake and lactate release were determined by using standard enzymatic test kits. The adenylate energy charge decreased with time under hypoxia in all cell lines. The decrease was most pronounced shortly after the treatment had been initiated and then tapered off. BEX-c and SAX-c showed a significantly higher adenylate energy charge under hypoxic conditions than did COX-c and WIX-c whether the pHe was 7.4, 7.0 or 6.6, showing that tumours can differ in the ability to avoid energy deprivation during microenvironmental stress. There was no correlation between the adenylate energy charge and the rates of glucose uptake and lactate release. Intertumour heterogeneity in the ability to withstand energy deprivation in an oxygen-deficient microenvironment cannot therefore be attributed mainly to differences in the capacity of the tumour cells to generate energy by anaerobic metabolism. The data presented here suggest that the heterogeneity is rather caused by differences in the capacity of the tumour cells to reduce the rate of energy consumption when exposed to hypoxia.


British Journal of Cancer (1997) 76(4), 421–428
© 1997 Cancer Research Campaign

### Energy metabolism in human melanoma cells under hypoxic and acidic conditions in vitro

**R Skøyum, K Eide, K Berg and EK Rofstad**

Institute for Cancer Research, The Norwegian Radium Hospital, Montebello, 0310 Oslo, Norway

**Summary** The response to treatment and the malignant progression of tumours are influenced by the ability of the tumour cells to withstand severe energy deprivation during prolonged exposure to hypoxia at normal or low extracellular pH ($pH_e$). The objective of the present work was to demonstrate intertumour heterogeneity under conditions of microenvironment-induced energy deprivation and to investigate whether the heterogeneity can be attributed to differences in the capacity of the tumour cells to generate energy in an oxygen-deficient microenvironment. Cultures of four human melanoma cell lines (BEX-c, COX-c, SAX-c, WIX-c) were exposed to hypoxia in vitro at $pH_e$ 7.4, 7.0 or 6.6 for times up to 31 h by using the steel-chamber method. High-performance liquid chromatography was used to assess adenylate energy charge as a function of exposure time. Cellular rates of glucose uptake and lactate release were determined by using standard enzymatic test kits. The adenylate energy charge decreased with time under hypoxia in all cell lines. The decrease was most pronounced shortly after the treatment had been initiated and then tapered off. BEX-c and SAX-c showed a significantly higher adenylate energy charge under hypoxic conditions than did COX-c and WIX-c whether the $pH_e$ was 7.4, 7.0 or 6.6, showing that tumours can differ in the ability to avoid energy deprivation during microenvironmental stress. There was no correlation between the adenylate energy charge and the rates of glucose uptake and lactate release. Intertumour heterogeneity in the ability to withstand energy deprivation in an oxygen-deficient microenvironment cannot therefore be attributed mainly to differences in the capacity of the tumour cells to generate energy by anaerobic metabolism. The data presented here suggest that the heterogeneity is rather caused by differences in the capacity of the tumour cells to reduce the rate of energy consumption when exposed to hypoxia.

**Keywords**: adenylate energy charge; energy metabolism; extracellular pH; hypoxia; melanoma

The metabolic microenvironment of tumours differs substantially from that of the corresponding normal tissue (Vaupel et al, 1989). Many tumours develop hypoxic regions and regions with low extracellular pH ($pH_e$) during growth (Wike-Hooley et al, 1984; Stone et al, 1993). Two types of hypoxia have been recognized in tumours: chronic hypoxia, resulting from limitations in oxygen diffusion; and acute hypoxia, resulting from transient cessations in blood flow (Horsman, 1995). Low $pH_e$ in tumours is mainly a result of increased rate of glycolysis and poor vascularization, leading to inadequate removal of $H^+$ (Vaupel et al, 1989). The rate of glycolysis is up-regulated in hypoxic tumour cells (Robin et al, 1984), and as anaerobic glycolysis leads to accumulation of lactic acid (Busa and Nuccitelli, 1984; Wike-Hooley et al, 1984; Vaupel et al, 1989) the $pH_e$ might be particularly low in the hypoxic regions of tumours (Kallinowski and Vaupel, 1988; Tannock and Rotin, 1989; Martin and Jain, 1994).

Tumour treatment response depends on the metabolic microenvironment of the tumour cells (Wike-Hooley et al, 1984; Sutherland et al, 1988). Hypoxic tumour cells are resistant to radiation therapy (Coleman, 1988) and some forms of chemotherapy (Teicher, 1994). The radiation dose required to inactivate tumour cells under hypoxic conditions is 2.5–3.0 times higher than that required under aerobic conditions (Stone et al, 1993). Tumour cells at low $pH_e$ also show increased radiation resistance, although the increase is much less than that due to hypoxia (Tannock and Rotin, 1989; Durand, 1991). The cytotoxic activity of chemotherapeutic drugs is increased, unchanged or decreased at low $pH_e$, depending on the tumour cells and the mechanism of action of the drug (Tannock and Rotin, 1989; Teicher, 1994).

The malignant progression of tumours is also influenced by the metabolic microenvironment of the tumour cells (Hill, 1990). The expression of several specific genes involved in the malignant progression is increased in hypoxic tumour cells and tumour cells at low $pH_e$ (Brown and Giaccia, 1994; Dachs and Stratford, 1996). Tumour cells can show increased metastatic potential following transient exposure to hypoxia (Young et al, 1988; Young and Hill, 1990) or low $pH_e$ (Schlappack et al, 1991). Moreover, hypoxia followed by reoxygenation can lead to increased resistance to some chemotherapeutic drugs (Rice et al, 1987; Luk et al, 1990; Sanna and Rofstad, 1994) and to the development of new cell subpopulations showing increased DNA content (Rice et al, 1986; Wilson et al, 1989) or a doubling of the number of chromosomes (Rofstad et al, 1996*a*).

The probability of microenvironment-induced tumour treatment failure or malignant progression is influenced by the ability of the tumour cells to withstand severe energy deprivation during prolonged exposure to hypoxia at normal or low $pH_e$ (Coleman, 1988; Tannock and Rotin, 1989; Vaupel et al, 1989; Hill, 1990). The energy status of tissues is controlled by the balance between the rates of ATP formation and utilization. The ATP formation is reduced markedly in tumour cells in a hypoxic microenvironment,

*Received 5 November 1996*
*Revised 5 February 1997*
*Accepted 10 February 1997*

*Correspondence to:* EK Rofstad, Department of Biophysics, Institute for Cancer Research, The Norwegian Radium Hospital, Montebello, 0310 Oslo, Norway mainly because of inhibition of respiration but also because of inhibition of glycolysis by low $pH_e$ (Rotin et al, 1986; Gerweck et al, 1993). ATP utilization is subsequently reduced, leading to decreased DNA and protein synthesis and cessation of cell proliferation (Born et al, 1976; Heacock and Sutherland, 1990; Casciari et al, 1992). The rate of energy deprivation during exposure to hypoxia at normal or low $pH_e$ might thus differ between tumours because of differences in the capacity of the tumour cells to generate energy and/or to reduce the energy consumption.

The main purpose of the work reported here was to demonstrate intertumour heterogeneity under conditions of microenvironment-induced energy deprivation and to investigate whether the heterogeneity can be attributed to differences in the capacity of the tumour cells to generate energy in an oxygen-deficient microenvironment. Cell cultures of four human melanoma lines were exposed to hypoxia in vitro at $pH_e$ 7.4, 7.0 or 6.6, and adenylate energy charge was measured as a function of exposure time. As glucose is the main energy source of tumour cells under hypoxic conditions (Wike-Hooley et al, 1984; Vaupel et al, 1989), the rates of glucose uptake and lactate release were used as measures of energy generation.

### MATERIALS AND METHODS

### Cell lines

Four human melanoma cell lines (BEX-c, COX-c, SAX-c, WIX-c) were included in the study (Rofstad et al, 1991). The cell lines were maintained in monolayer culture in RPMI-1640 medium (25 mM Hepes and L-glutamine) supplemented with 13% fetal calf serum, 250 mg $l^{-1}$ penicillin and 50 mg $l^{-1}$ streptomycin. The cultures were incubated at 37°C in a humidified atmosphere of 5% carbon dioxide in air and subcultured once a week by trypsinization (treatment with 0.05% trypsin/0.02% EDTA solution at 37°C for 2 min). All solutions were purchased from Life Sciences Technology, UK. The cell lines were verified to be free from *Mycoplasma* contamination by using the Hoechst fluorescence and the mycotrin methods.

### Exposure to hypoxia

Monolayer cell cultures growing in glass dishes were exposed to hypoxia by using the steel-chamber method (Sanna and Rofstad, 1994). The cultures were incubated at 37°C in a humidified atmosphere of 5% carbon dioxide in air for 24 h before the hypoxia treatment. The culture medium was removed and replaced by fresh medium before the cells were exposed to hypoxia. The medium used during the hypoxia treatment was supplemented with 1.1 mg $ml^{-1}$ sodium bicarbonate and adjusted to a pH of 7.4, 7.0 or 6.6 using sodium hydroxide or hydrochloric acid. Before use, it was flushed with 5% carbon dioxide in air for 2 h and then the pH was readjusted (Boyer et al, 1993). The glass dishes were kept in air-tight steel chambers during the hypoxia treatment. The steel chambers were flushed with a humidified, highly purified gas mixture consisting of 95% nitrogen and 5% carbon dioxide at a flow rate of 5 1 $min^{-1}$. Measurements showed that the concentration of oxygen in the medium was less than 10 p.p.m. after 30 min of flushing. Control cultures were flushed with humidified 5% carbon dioxide in air. Separate dishes were used for measurement of adenylate energy charge and concentrations of glucose and lactate. After medium samples had been collected for measurement of glucose and lactate concentrations, the cells were detached from the dishes by trypsinization and counted by using a haemocytometer and a phase-contrast microscope.

**Figure 1** Extracellular pH in hypoxic cell cultures vs time under hypoxia. The cells were cultured in medium adjusted to a pH of 7.4 (○), 7.0 (■) or 6.6 (△) immediately before the hypoxia exposure. The curves refer to single experiments with BEX-c. Similar curves were achieved in all experiments, irrespective of cell line

**Figure 2** Relative cell number vs time for aerobic (○) and hypoxic (●) cell cultures. The curves refer to single experiments with BEX-c at $pH_e$ 7.4. Similar curves were achieved in all experiments, irrespective of cell line and $pH_e$

© Cancer Research Campaign 1997

### Measurements of rates of glucose uptake and lactate release

Cellular rates of glucose uptake and lactate release were determined by using standard enzymatic test kits (Boehringer Mannheim, Mannheim, Germany) and spectrophotometric assays (Casciari et al, 1992). The glucose and lactate concentrations in the media of aerobic and hypoxic cell cultures were measured as a function of time. The rates of the concentration changes were divided by the logarithmic mean cell number to obtain rates of glucose uptake and lactate release in terms of mol per cell per second (Rofstad et al, 1996*b*).

### Measurement of adenylate energy charge

The adenylate phosphates were separated by high-performance liquid chromatography and quantified at 254 nm. The cells were lysed in acetonitrile ($CH_3CN$), scraped off the dishes and dried in nitrogen. Low-strength buffer (100 mM potassium dihydrogen phosphate, 1.5% acetonitrile, 0.08% tetrabutylammonium bromide ($C_{16}H_{36}NBr$), pH 5.0) was added before centrifugation and collection of the supernatant. The elution was performed with 80% low-strength buffer and 20% high-strength buffer (150 mM potassium dihydrogen phosphate, 10% acetonitrile, 0.08% tetrabutyl-ammonium bromide, pH 5.0) for 10 min, then with a linear

**Figure 3** Medium concentration of glucose (A and B) and lactate (C and D) in hypoxic cell cultures vs time under hypoxia. The curves refer to single experiments with BEX-c at $pH_e$ 7.4 or 6.6. Similar curves were achieved in all experiments, irrespective of cell line

© Cancer Research Campaign 1997

**Figure 4** Rates of glucose uptake and lactate release in BEX-c, COX-c, SAX-c and WIX-c under aerobic and hypoxic conditions. The experiments were performed at $pH_e$ 7.4. Columns and bars represent mean values ± s.d. of 4–9 independent experiments. □, Rate of glucose uptake; ■, rate of lactate release gradient to 100% high-strength buffer for 10 min and finally with the high-strength buffer for 10 min. The separation was carried out with a Supelcosil LC-18-T 5-µm cartridge (Supelco, SA, Crans, Switzerland). The flow rate was 1.0 ml $min^{-1}$. Adenylate energy charge (AEC) was calculated from peak areas:

$$AEC = ([ATP] + 1/2\ [ADP])/([ATP] + [ADP] + [AMP])$$

### Statistical analysis

Results are presented as individual values or as arithmetic means ± standard deviation (s.d.). Statistical comparisons of mean values were performed under conditions of normality and equal variance by using the Student's *t*-test for single comparisons and one-way analysis of variance and the Student–Newman–Keuls test for multiple comparisons. Paired tests were performed where appropriate. All *P*-values were determined from two-sided tests. A significance criterion of $P < 0.05$ was used. The statistical analysis was performed using SigmaStat statistical software (Jandel Scientific, Erkrath, Germany).

### RESULTS

Cell cultures were exposed to hypoxia at $pH_e$ 7.4, 7.0 or 6.6 for 3–31 h. In this time interval, the $pH_e$ decreased by 0.2 pH units at $pH_e$ 7.4 and 7.0 and by 0.4 pH units at $pH_e$ 6.6 (Figure 1). The cells did not proliferate during the hypoxia treatment. The number of cells per dish increased exponentially with time in aerobic control cultures and stayed unchanged in hypoxic cultures (Figure 2). The cells remained attached to the glass surface during the hypoxia treatment. The fraction of trypan blue excluding cells after 31 h of hypoxia was higher than 95% in all cell lines, independent of the $pH_e$.

The glucose concentration in the medium of hypoxic cell cultures decreased linearly with time at $pH_e$ 7.4 and 7.0 and, correspondingly, the lactate concentration increased linearly with time (Figure 3). In contrast, the glucose and lactate concentration curves were not linear at $pH_e$ 6.6: they showed a break between 10 and 14 h (Figure 3). The rates of glucose and lactate concentration changes were determined by linear regression analysis. Single values were derived from each data set at $pH_e$ 7.4 and 7.0. Two values were derived at $pH_e$ 6.6: one early value based on analysis

© Cancer Research Campaign 1997

**Figure 5** Rates of glucose uptake and lactate release in BEX-c, COX-c, SAX-c and WIX-c under hypoxic conditions. The experiments were performed at $pH_e$ 7.4, 7.0 or 6.6. Columns and bars represent mean values ± s.d. of four or five independent experiments. □, Rate of glucose uptake; ■, rate of loctate release of the data in the time interval 0–10 h and one late value based on analysis of the data in the time interval 14–31 h. The rates of the glucose and lactate concentration changes in the medium of aerobic cell cultures were determined by linear regression analysis of data pertaining to a 6-h time interval (figure not shown).

The rates of glucose uptake and lactate release in BEX-c, COX-c, SAX-c and WIX-c under hypoxic conditions are compared with those under aerobic conditions in Figure 4. The experiments were performed at $pH_e$ 7.4. BEX-c showed lower rates of glucose uptake and lactate release than COX-c, SAX-c and WIX-c under aerobic conditions ($P < 0.01$). The rates of glucose uptake and lactate release were higher under hypoxic conditions than under aerobic conditions in all cell lines ($P < 0.05$). The magnitude of the hypoxia-induced glycolysis up-regulation was not significantly different in the four cell lines.

Figure 5 shows the rates of glucose uptake and lactate release in BEX-c, COX-c, SAX-c and WIX-c under hypoxic conditions at $pH_e$ 7.4, 7.0 and 6.6 (early and late). The glucose metabolism differed significantly between some of the cell lines at $pH_e$ 7.4, 7.0 and 6.6 (early). Thus, the rates of glucose uptake and lactate release were higher in COX-c, SAX-c and WIX-c than in BEX-c at $pH_e$ 7.4 ($P < 0.01$) and 7.0 ($P < 0.01$). SAX-c showed higher rates of glucose uptake and lactate release than BEX-c, COX-c and WIX-c at $pH_e$ 6.6 (early) ($P < 0.05$). Significant differences between the cell lines were not detected $pH_e$ 6.6 (late).

The glucose metabolism was similar at $pH_e$ 7.4 and 7.0 and inhibited at $pH_e$ 6.6 (Figure 5). Thus, the rate of glucose uptake was lower at $pH_e$ 6.6 (early and late) than at $pH_e$ 7.4 and 7.0 in all cell lines ($P < 0.05$). All lines also showed a lower rate of lactate release at $pH_e$ 6.6 (late) than at $pH_e$ 7.4 and 7.0 ($P < 0.01$). However, the rate of lactate release at $pH_e$ 6.6 (early) was not significantly different from that at $pH_e$ 7.4 and 7.0 in any of the lines. It should also be noticed that at $pH_e$ 6.6 (early), the rate of lactate release was higher than the rate of glucose uptake by a factor larger than 2.0.

© Cancer Research Campaign 1997

**Figure 6** Chromatograms showing the separation and the relative concentrations of AMP, ADP and ATP. The number at the peaks represents the exact elution time. The chromatograms refer to BEX-c exposed to hypoxia at $pH_e$ 7.4 for 0, 10, 18 or 31 h. Similar chromatograms were achieved in all experiments, irrespective of cell line and $pH_e$

The adenylate energy charge of the cell lines was 0.94 ± 0.01 (BEX-c), 0.92 ± 0.02 (COX-c), 0.93 ± 0.03 (SAX-c) and 0.93 ± 0.02 (WIX-c) under aerobic conditions at $pH_e$ 7.4. Chromatograms showing that the adenylate energy charge decreased with time under hypoxia are presented in Figure 6. The ATP peak was reduced and the AMP and ADP peaks were enhanced after protracted hypoxia treatments. The adenylate energy charge followed biphasic curves; the decrease was most pronounced shortly after the hypoxia treatment had been initiated and then tapered off (Figure 7). The magnitude of the hypoxia-induced decrease differed significantly between some of the cell lines. Thus, 6–31 h after the initiation of treatment, the adenylate energy charge was higher in BEX-c and SAX-c than in COX-c and WIX-c at $pH_e$ 7.4 ($P < 0.05$), 7.0 ($P < 0.05$) and 6.6 ($P < 0.05$). The adenylate energy charge in BEX-c was not significantly different from that in SAX-c and the adenylate energy charge in COX-c was not significantly different from that in WIX-c at any of these $pH_e$ values. Adenylate energy charge curves were therefore fitted to the combined BEX-c and SAX-c data and to the combined COX-c and WIX-c data in Figure 7. The adenylate energy charge was independent of the $pH_e$; the curves at $pH_e$ 7.4, 7.0 and 6.6 were not significantly different in any of the cell lines.

### DISCUSSION

The adenylate energy charge of BEX-c, COX-c, SAX-c and WIX-c decreased during exposure to hypoxia. Hypoxia resulted in up-regulated glycolysis in all four lines. However, the up-regulation was not sufficiently large to compensate for the loss in energy generation resulting from the inhibition of the oxidative pathway. The effects of hypoxia on glycolysis and adenylate energy charge reported here for human melanoma cells are thus consistent with those reported previously for rodent tumour cells and other histological types of human tumour cells (Rotin et al, 1986; Heacock and Sutherland, 1990; Casciari et al, 1992; Gerweck et al, 1992).

The hypoxia-induced decrease in adenylate energy charge differed significantly between the four cell lines. Thus, BEX-c and SAX-c showed a higher adenylate energy charge under hypoxic conditions than did COX-c and WIX-c, whether the $pH_e$ was 7.4,

**Figure 7** Adenylate energy charge vs time under hypoxia for BEX-c (○), COX-c (■), SAX-c (△) and WIX-c (▼). The experiments were performed at $pH_e$ 7.4, 7.0 or 6.6. Points and bars represent mean values ± s.d. of four or five independent experiments. Curves were fitted to the data by regression analysis. The two curves in each panel represent the best common fit to the BEX-c and SAX-c data and the best common fit to the COX-c and WIX-c data

*© Cancer Research Campaign 1997*

7.0 or 6.6. This observation suggests that tumour cells can differ substantially in their ability to avoid energy deprivation during microenvironmental stress. As all cell lines studied here were of melanoma origin, significant intertumour heterogeneity in microenvironment-induced energy deprivation might occur even within single histological types of tumours.

The differences between the cell lines in adenylate energy charge during exposure to hypoxia cannot be attributed mainly to differences in the capacity of the cells to generate energy by anaerobic metabolism, because there was no correlation between the adenylate energy charge and the rates of glucose uptake and lactate release. Thus, the adenylate energy charge was similar for BEX-c and SAX-c, irrespective of the $pH_e$, whereas BEX-c showed lower rates of glucose uptake and lactate release than SAX-c, except at $pH_e$ 6.6 (late). SAX-c showed a higher adenylate energy charge than COX-c and WIX-c at $pH_e$ 7.4 and 7.0, whereas the rates of glucose uptake and lactate release were similar. Moreover, the adenylate energy charge at $pH_e$ 6.6 was similar to that at $pH_e$ 7.4 and 7.0 in all cell lines, whereas the rates of glucose uptake were down-regulated at $pH_e$ 6.6.

The adenylate energy charge of tumour cells subjected to microenvironmental stress is controlled by the balance between the rates of energy generation and energy consumption (Calderwood et al, 1985; Rotin et al, 1986; Gerweck et al, 1992). Exposure of cells to hypoxia leads to continuously decreasing rates of DNA and protein synthesis and cessation of cell proliferation (Born et al, 1976; Shrieve et al, 1983; Pettersen et al, 1986; Heacock and Sutherland, 1990). Cell lines might differ in the capacity to regulate and turn off these energy-requiring processes (Gerweck et al, 1992). Consequently, the higher adenylate energy charge in BEX-c and SAX-c than in COX-c and WIX-c might have resulted from a faster hypoxia-induced decrease in the energy consumption in BEX-c and SAX-c than in COX-c and WIX-c. This interpretation is consistent with the biphasic shape of the adenylate energy charge curves. The decrease in adenylate energy charge was most pronounced shortly after the hypoxia treatment had been initiated and then tapered off with time.

The $pH_e$ of most tumours in vivo is within the range 6.6–7.4 (Wike-Hooley et al, 1984; Tannock and Rotin, 1989; Vaupel et al, 1989). Experimental studies have suggested that the energy status is particularly low in hypoxic tumour cells at $pH_e$ in the lower part of this range, because of inhibited glycolysis owing to the low $pH_e$ (Halperin et al, 1969; Rotin et al, 1986; Casciari et al, 1992; Gerweck et al, 1993). However, the adenylate energy charge at $pH_e$ 6.6 was similar to that at $pH_e$ 7.4 and 7.0 in hypoxic cultures of the cell lines studied here, even though the rate of glucose uptake was lower at $pH_e$ 6.6, than at $pH_e$ 7.4 and 7.0. It is possible that the melanoma cells used an intracellular energy source at $pH_e$ 6.6, e.g. a stock of glycogen, to compensate for the reduced rate of glucose uptake. This suggestion is consistent with the observation that the rate of lactate release was higher than the rate of glucose uptake by a factor larger than 2.0 during the first 10 h of the hypoxia treatment at $pH_e$ 6.6. The rate of lactate release corresponded to the rate of glucose uptake 14–31 h after the treatment had been initiated (Figure 5), indicating that the intracellular energy source had been exhausted. The rate of energy consumption might have been adjusted to the reduced rate of energy generation by then, and hence, the adenylate energy charge at $pH_e$ 6.6 was similar to that at $pH_e$ 7.4 and 7.0 in hypoxic BEX-c, COX-c, SAX-c and WIX-c cultures.

In summary, hypoxia and low $pH_e$ might cause resistance to treatment and promote the malignant progression of tumours, provided that the tumour cells have the ability to avoid severe energy deprivation during prolonged microenvironmental stress. The present study suggests that tumours differ in this ability and that the differences can be attributed to differences in the capacity to turn off energy-requiring processes in an oxygen-deficient microenvironment rather than to differences in the capacity to generate energy by anaerobic metabolism.

### ACKNOWLEDGEMENT

Financial support was received from The Norwegian Cancer Society.

### REFERENCES

Born R, Hug O and Trott KR (1976) The effect of prolonged hypoxia on the growth and viability of Chinese hamster cells. *Int J Radiat Biol* **1**: 687–697

Boyer MJ, Bernard M, Hedley DW and Tannock IF (1993) Regulation of intracellular pH in subpopulations of cells derived from spheroids and solid tumours. *Br J Cancer* **68**: 890–897

Brown JM and Giaccia AJ (1994) Tumor hypoxia: the picture has changed in the 1990s. *Int J Radiat Biol* **65**: 95–102

Busa WB and Nuccitelli R (1984) Metabolic regulation via intracellular pH. *Am J Physiol* **246**: R409–R438.

Calderwood SK, Bump EA, Stevenson MA, van Kersen I and Hahn GM (1985) Investigation of adenylate energy charge, phosphorylation potential, and ATP concentration in cells stressed with starvation and heat. *J Cell Physiol* **124**: 261–268

Casciari JJ, Sotirchos SV and Sutherland RM (1992) Variation in tumor cell growth rates and metabolism with oxygen concentration, glucose concentration, and extracellular pH. *J Cell Physiol* **151**: 386–394

Coleman CN (1988) Hypoxia in tumors: a paradigm for the approach to biochemical and physiologic heterogeneity. *J Natl Cancer Inst* **80**: 310–317

Dachs GU and Stratford IJ (1996) The molecular response of mammalian cells to hypoxia and the potential for exploitation in cancer therapy. *Br J Cancer* **74**: S126–S132

Durand RE (1991) Keynote address: the influence of microenvironmental factors on the activity of radiation and drugs. *Int J Radiat Oncol Biol Phys* **20**: 253–258

Gerweck LE, Koutcher JA, Zaidi ST and Seneviratne T (1992) Energy status in the murine FSaII and MCaIV tumors under aerobic and hypoxic conditions: an in-vivo and in-vitro analysis. *Int J Radiat Oncol Biol Phys* **23**: 557–561

Gerweck LE, Seneviratne T and Gerweck KK (1993) Energy status and radiobiological hypoxia at specified oxygen concentrations. *Radiat Res* **135**: 69–74

Halperin ML, Conners HP, Relman AS and Karnovsky ML (1969) Factors that control the effect of pH on glycolysis in leukocytes. *J Biol Chem* **244**: 384–390

Heacock CS and Sutherland RM (1990) Enhanced synthesis of stress proteins caused by hypoxia and relation to altered cell growth and metabolism. *Br J Cancer* **62**: 217–225

Hill RP (1990) Tumor progression: potential role of unstable genomic changes. *Cancer Metastasis Rev* **9**: 137–147

Horsman MR (1995) Nicotinamide and other benzamide analogs as agents for overcoming hypoxic cell radiation resistance in tumours. *Acta Oncol* **34**: 571–587

Kallinowski F and Vaupel P (1988) pH distributions in spontaneous and isotransplanted rat tumours. *Br J Cancer* **58**: 314–321

Luk CK, Veinot-Drebot L, Tjan E and Tannock IF (1990) Effect of transient hypoxia on sensitivity to doxorubicin in human and murine cell lines. *J Natl Cancer Inst* **82**: 684–692

Martin GR and Jain RK (1994) Noninvasive measurement of interstitial pH profiles in normal and neoplastic tissue using fluorescence ratio imaging microscopy. *Cancer Res* **54**: 5670–5674

Pettersen EO, Juul NO and Rønning ØW (1986) Regulation of protein metabolism of human cells during and after acute hypoxia. *Cancer Res* **46**: 4346–4351

Rice GC, Hoy C and Schimke RT (1986) Transient hypoxia enhances the frequency of dihydrofolate reductase gene amplification in Chinese hamster ovary cells. *Proc Natl Acad Sci USA* **83**: 5978–5982

Rice GC, Ling V and Schimke RT (1987) Frequencies of independent and simultaneous selection of Chinese hamster cells for methotrexate and doxorubicin (adriamycin) resistance. *Proc Natl Acad Sci USA* **84**: 9261–9264

© Cancer Research Campaign 1997

Robin ED, Murphy BJ and Theodore J (1984) Coordinate regulation of glycolysis by hypoxia in mammalian cells. *J Cell Physiol* **118**: 287–290

Rofstad EK, Wahl A, Hystad ME, Nesland JM and Stokke T (1991) Establishment in monolayer culture and characterization of four human melanoma cell lines. *Virchows Arch B Cell Pathol* **60**: 189–197

Rofstad EK, Johnsen NM and Lyng H (1996*a*) Hypoxia-induced tetraploidisation of a diploid human melanoma cell line in vitro. *Br J Cancer* **74**: S136–S139

Rofstad EK, Eide K, Skøyum R, Hystad ME and Lyng H (1996*b*) Apoptosis, energy metabolism, and fraction of radiobiologically hypoxic cells: a study of human melanoma multicellular spheroids. *Int J Radiat Biol* **70**: 241–249

Rotin D, Robinson B and Tannock IF (1986) Influence of hypoxia and an acidic environment on the metabolism and viability of cultured cells: potential implications for cell death in tumors. *Cancer Res* **46**: 2821–2826

Sanna K and Rofstad EK (1994) Hypoxia-induced resistance to doxorubicin and methotrexate in human melanoma cell lines in vitro. *Int J Cancer* **58**: 258-262

Schlappack OK, Zimmermann A and Hill RP (1991) Glucose starvation and acidosis: effect on experimental metastatic potential, DNA content and MTX resistance of murine tumour cells. *Br J Cancer* **64**: 663–670

Shrieve DC, Deen DF and Harris JW (1983) Effect of extreme hypoxia on the growth and viability of EMT6/SF mouse tumor cells in vitro. *Cancer Res* **43**: 3521–3527

Stone HB, Brown JM, Phillips TL and Sutherland RM (1993) Oxygen in human tumors: correlations between methods of measurement and response to therapy. *Radiat Res* **136**: 422–434

Sutherland RM, Rasey JS and Hill RP (1988) Tumor biology. *Am J Clin Oncol* **11**: 253–274

Tannock IF and Rotin D (1989) Acid pH in tumors and its potential for therapeutic exploitation. *Cancer Res* **49**: 4373–4384

Teicher BA (1994) Hypoxia and drug resistance. *Cancer Metastasis Rev* **13**: 139–168

Vaupel P, Kallinowski F and Okunieff P (1989) Blood flow, oxygen and nutrient supply, and metabolic microenvironment of human tumors: a review. *Cancer Res* **49**: 6449–6465

Wike-Hooley JL, Haveman J and Reinhold HS (1984) The relevance of tumour pH to the treatment of malignant diseases. *Radiother Oncol* **2**: 343–366

Wilson RE, Keng PC and Sutherland RM (1989) Changes in growth characteristics and macromolecular synthesis on recovery from severe hypoxia. *Br J Cancer* **61**: 14–21

Young SD and Hill RP (1990) Effects of reoxygenation on cells from hypoxic regions of solid tumors: anticancer drug sensitivity and metastatic potential. *J Natl Cancer Inst* **82**: 371–380

Young SD, Marshall RS and Hill RP (1988) Hypoxia induces DNA overreplication and enhances metastatic potential of murine tumor cells. *Proc Natl Acad Sci USA* **85**: 9533–9537

*© Cancer Research Campaign 1997*